# Comparability of clinical trials and spontaneous reporting data regarding COVID-19 vaccine safety

**DOI:** 10.1038/s41598-022-13809-7

**Published:** 2022-06-29

**Authors:** Chongliang Luo, Jingcheng Du, Adam Cuker, Ebbing Lautenbach, David A. Asch, Gregory A. Poland, Cui Tao, Yong Chen

**Affiliations:** 1 Department of Biostatistics, Epidemiology and Informatics, University of Pennsylvania, Philadelphia, PA 19104 USA; 2 Division of Public Health Sciences, Washington University School of Medicine in St. Louis, St. Louis, MO USA; 3 School of Biomedical Informatics, The University of Texas Health Science Center at Houston, Houston, TX USA; 4 Department of Medicine and Department of Pathology and Laboratory Medicine, Perelman School of Medicine, University of Pennsylvania, Philadelphia, PA USA; 5 Division of Infectious Diseases, Department of Medicine, Perelman School of Medicine, University of Pennsylvania, Philadelphia, PA USA; 6 Center for Clinical Epidemiology and Biostatistics, Perelman School of Medicine, University of Pennsylvania, Philadelphia, PA USA; 7 Division of General Internal Medicine, University of Pennsylvania, Philadelphia, PA USA; 8 Leonard Davis Institute of Health Economics, Philadelphia, PA USA; 9 Mayo Clinic Vaccine Research Group, Mayo Clinic, Rochester, MN USA

**Keywords:** Vaccines, Signs and symptoms

## Abstract

Severe adverse events (AEs) after COVID-19 vaccination are not well studied in randomized controlled trials (RCTs) due to rarity and short follow-up. To monitor the safety of COVID-19 vaccines (“Pfizer” vaccine dose 1 and 2, “Moderna” vaccine dose 1 and 2, and “Janssen” vaccine single dose) in the U.S., especially regarding severe AEs, we compare the relative rankings of these vaccines using both RCT and the Vaccine Adverse Event Reporting System (VAERS) data. The risks of local and systemic AEs were assessed from the three pivotal COVID-19 vaccine trials and also calculated in the VAERS cohort consisting of 559,717 reports between December 14, 2020 and September 17, 2021. AE rankings of the five vaccine groups calculated separately by RCT and VAERS were consistent, especially for systemic AEs. For severe AEs reported in VAERS, the reported risks of thrombosis and GBS after Janssen vaccine were highest. The reported risk of shingles after the first dose of Moderna vaccine was highest, followed by the second dose of the Moderna vaccine. The reported risk of myocarditis was higher after the second dose of Pfizer and Moderna vaccines. The reported risk of anaphylaxis was higher after the first dose of Pfizer vaccine. Limitations of this study are the inherent biases of the spontaneous reporting system data, and only including three pivotal RCTs and no comparison with other active vaccine safety surveillance systems.

## Introduction

The COVID-19 vaccines (BNT162b2 mRNA vaccine, or “Pfizer”, mRNA-1273 vaccine, or “Moderna”, and Ad26.COV2.S vaccine, or “Janssen”) are critical components of the response to the pandemic globally. The efficacy and safety of the three COVID-19 vaccines administered in the US were reported in a series of randomized controlled trials (RCTs)^1–4^. These RCTs reported risks of mild and common local and systemic adverse events (AEs). For some other severe but rare AEs, risks were not explicitly estimated in the RCTs, likely due to few cases or short follow-up. However, the consequence of these AEs may be more substantial. For example, complications such as thrombosis^5^ or Guillain–Barré syndrome (GBS) may occur after vaccination^6,7^. Despite their severity, the incidence of these rare AEs are difficult to estimate in RCTs.

Post-marketing surveillance of COVID-19 vaccines offers larger sample sizes from which to estimate rare AEs, but may create bias because the data are collected less systematically than in RCTs. For example, the Vaccine Adverse Event Reporting System (VAERS), maintained by the Centers for Disease Control and Prevention (CDC) and FDA, provides real-world post-marketing evidence of vaccine safety and has been used to monitor safety of vaccines, including COVID-19 vaccines^5,8–10^. But because it organically accepts reports from healthcare professionals as well as any member of the population, it is unclear whether VAERS reports can replace or anticipate results from more systematic assessments, or what biases may be present.

In this paper, we compare the relative rankings of three COVID-19 vaccines available in the U.S. using both RCT and VAERS data to assess how consistently these data sources perform. We first analyzed common local (i.e. pain, erythema and swelling) and systemic (i.e. headache, fever, chill, fatigue, nausea/vomiting, arthralgia and myalgia) AE signals after COVID-19 vaccines from VAERS data and then compared the results with RCT data. We then “extrapolated” the consistency and further ranked the COVID-19 vaccines regarding rare but severe AEs, including shingles, hearing impairment, thrombosis, facial paralysis, anaphylaxis, pulmonary embolism, myocarditis, and GBS. Though these AEs have drawn more public attention and may result in more severe outcomes, they were not explicitly evaluated in the RCTs due to the rarity of events and/or short follow-up.

## Results

We extracted 569,072 reports submitted to VAERS related to COVID-19 vaccines from December 14, 2020 to September 17, 2021. After excluding 1952 entries reporting co-administration with other vaccines, 1198 reports with an unknown manufacturer, and 6297 reports without associated symptoms; 559,717 reports were used for analysis, including 237,735 for the Pfizer, 270,754 for the Moderna, and 51,228 for the Janssen vaccines. The characteristics of these VAERS reports are presented in Table 1.

**Table 1:** Characteristics of the U.S. VAERS reports after COVID-19 vaccines.

Vaccine	Pfizer	Moderna	Janssen
Dates of vaccination, range	12/14/2020–9/17/2021	12/14/2020–9/17/2021	03/02/2021–9/17/2021
Total administered^a^ (M)	224 (P1 = 114, P2 = 100)	151 (M1 = 83, M2 = 68)	15
Total reported: N (%)	237,735 (100)	270,754 (100)	51,228 (100)
**Age**			
< 18	13,738 (5.8)	4394 (1.6)	728 (1.4)
18–59	144,841 (60.9)	144,822 (53.5)	32,662 (63.8)
60 +	59,425 (25.0)	95,895 (35.4)	8894 (17.4)
Unknown	19,731 (8.3)	25,643 (9.5)	8944 (17.5)
**Sex**			
Female	163,797 (68.9)	192,018 (70.9)	30,250 (59)
Male	68,964 (29)	68,632 (25.3)	17,512 (34.2)
Unknown	4974 (2.1)	10,104 (3.7)	3466 (6.8)
**Onset days**			
0	99,639 (41.9)	99,327 (36.7)	18,736 (36.6)
1–7	81,344 (34.2)	94,388 (34.9)	11,892 (23.2)
8–42	23,284 (9.8)	43,587 (16.1)	5114 (10)
Unknown	33,468 (14.1)	33,452 (12.4)	15,486 (30.2)
**Dose**			
First	120,391 (50.6)	163,105 (60.2)	51,228 (100)
Second	82,570 (34.7)	71,230 (26.3)	0 (0)
Unknown	34,774 (14.6)	36,419 (13.5)	0 (0)
History of medication and health conditions^b^	106,424 (44.8)	124,591 (46)	20,552 (40.1)
Severe outcome^c^	21,881 (9.2)	17,703 (6.5)	4561 (8.9)

^a^The vaccine coverage data in the general population are obtained from CDC website^17^ as of September 17, 2021, numbers are rounded to millions. P1, P2 are two doses of Pfizer vaccines, similar for M1 and M2. ^b^Reported history of medications, allergies, short-term or acute illnesses, or chronic or long-standing health conditions. ^c^Include died, life-threatening, ER visit, hospitalized and disabled outcome.

VAERS reports were more common after administration of the Janssen vaccine (3.40 per 1000 doses) than Pfizer or Moderna vaccines (1.06 or 1.79 per 1000 doses, respectively). Fewer reports were related to the elderly population (60 +) after Janssen vaccine (17.4%), compared to Moderna (35.4%) and Pfizer (25.0%). Females were much more likely to submit VAERS reports than males, irrespective of vaccine type. The AEs reported were slightly more likely to occur on the same day as vaccination for the Pfizer vaccine (41.9%, compared to 36.7% and 36.6% for Moderna and Janssen respectively). More reports were related to the first dose than the second dose for Pfizer (50.6% vs 34.7%) and Moderna (60.2% vs 26.3%) vaccines.

**Figure 1:**
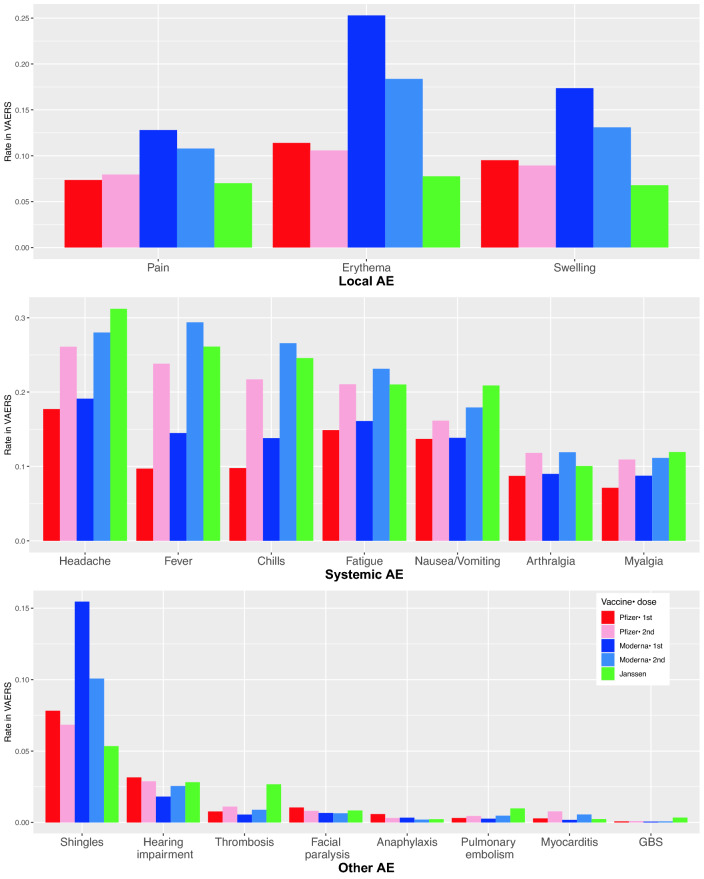
The reporting risks of AEs after COVID-19 vaccines reported in VAERS from 12/14/2020 to 09/17/2021. The denominators (numbers of VAERS reports on particular vaccine doses) are 83,877, 57,431, 101,383, 54,386 and 29,877 for Pfizer dose 1 and 2, Moderna dose 1 and 2, and Janssen vaccines respectively.

The main results are presented in Table 2, which contains the risks of AEs after COVID-19 vaccination as reported in either RCTs or VAERS, and the rank of vaccines and their consistency. The risks of AEs after COVID-19 vaccination as reported in VAERS are also shown in Fig. 1. As expected, the AE risks from systematically observed RCTs (the first rows) are substantially different than the AE risks spontaneously reported in the VAERS database (the second rows). However, the rank order of vaccine AEs are highly consistent between RCTs and VAERS, especially for systemic AEs. Specifically, for Pfizer and Moderna vaccines, the reporting risks of systemic AEs after the first dose are always lower than those after the second dose in either RCTs or VAERS. Results based on age groups are presented in Tables S1 and S2 in the Supplementary Materials. In general, the AE risks for elderly population is slightly lower than young population, while the rank of vaccines remains to be consistent for each of the two age groups.

**Table 2:** Risks (%) of various AEs related to the three COVID-19 vaccines (and doses) in the U.S. by randomized clinical trials (RCT) or vaccine adverse event reporting system (VAERS) data. Five groups of vaccines are compared, i.e. Pfizer-1st (P1), Pfizer-2nd (P2), Moderna-1st (M1), Moderna-2nd (M2) and Janssen (J). The denominator, i.e. sizes of safety set in RCTs are 9839, 9839, 15,168, 14,677 and 3356 for P1, P2, M1, M2 and J respectively, and numbers of reports in VAERS are 83,877, 57,431, 101,383, 54,386 and 29,877 for P1, P2, M1, M2 and J respectively. A rank correlation greater than 0 means the rank of vaccines by RCT and VAERS are consistent. The “other AEs” are not explicitly evaluated in RCTs.

Adverse events (AE)	Data source	AE risks (%) by vaccine groups					Rank					Rank correlation (Spearman’s $\rho$)
		P1	P2	M1	M2	J						
**Local AEs (joint evidence generated from RCTs and VAERS)**												
Pain	RCT	78	73.5	83.7	88.2	48.6	J	P2	P1	M1	M2	0.80
	VAERS	7.4	8	12.8	10.8	7.0	J	P1	P2	M2	M1	
Erythema	RCT	5	6.2	2.8	8.6	6.7	M1	P1	P2	J	M2	− 0.40
	VAERS	11.4	10.6	25.3	18.4	7.8	J	P2	P1	M2	M1	
Swelling	RCT	6.7	6.7	6.1	12.2	5.7	J	M1	P1	P2	M2	0.36
	VAERS	9.5	8.9	17.4	13.1	6.8	J	P2	P1	M2	M1	
**Systemic AEs (joint evidence generated from RCTs and VAERS)**												
Headache	RCT	35.5	47.9	32.7	58.6	39.3	M1	P1	J	P2	M2	0.60
	VAERS	17.7	26.1	19.1	28	31.2	P1	M1	P2	M2	J	
Fever	RCT	2.9	14.5	0.8	15.5	9.0	M1	P1	J	P2	M2	0.80
	VAERS	9.7	23.8	14.5	29.4	26.1	P1	M1	P2	J	M2	
Chills	RCT	11.4	31.1	8.3	44.2	NA	M1	P1	P2	M2	J	0.80
	VAERS	9.8	21.7	13.8	26.6	24.6	P1	M1	P2	J	M2	
Fatigue	RCT	42.3	56.9	37.2	65.3	39.0	M1	J	P1	P2	M2	0.70
	VAERS	14.9	21.1	16.1	23.1	21.0	P1	M1	J	P2	M2	
Nausea/vomiting	RCT	0.8	1.8	8.3	19	14.0	P1	P2	M1	J	M2	0.80
	VAERS	13.7	16.2	13.9	17.9	20.9	P1	M1	P2	M2	J	
Arthralgia (joint pain)	RCT	10.1	21.4	16.6	42.8	NA	P1	M1	P2	M2	J	1
	VAERS	8.7	11.8	9	11.9	10.1	P1	M1	J	P2	M2	
Myalgia (muscle pain)	RCT	18.6	34.4	22.7	58	34.0	P1	M1	J	P2	M2	0.70
	VAERS	7.1	10.9	8.7	11.1	11.9	P1	M1	P2	M2	J	
**Other AEs (unique evidence generated from VAERS)**												
Shingles	VAERS	7.8	6.8	15.5	10.1	5.3	J	P2	P1	M2	M1	
Hearing impairment	VAERS	3.1	2.9	1.8	2.5	2.8	M1	M2	J	P2	P1	N/A
Thrombosis	VAERS	0.8	1.1	0.5	0.9	2.7	M1	P1	M2	P2	J	
Facial paralysis	VAERS	1	0.8	0.7	0.6	0.8	M2	M1	J	P2	P1	
Anaphylaxis	VAERS	0.6	0.3	0.3	0.2	0.2	M2	J	P2	M1	P1	
Pulmonary embolism	VAERS	0.3	0.4	0.3	0.5	1	M1	P1	P2	M2	J	
Myocarditis	VAERS	0.3	0.8	0.2	0.5	0.2	M1	J	P1	M2	P2	
GBS	VAERS	0.1	0.1	0.1	0.1	0.3	M1	M2	P1	P2	J

Table 2 also reports the ranks of vaccines for other rare but severe AEs available in VAERS but not in RCT data. To this end, VAERS provided large-scale post-marketing evidence for comparing the three vaccines. For example, the risks of thrombosis and GBS after Janssen vaccine are the highest. Concerns about excessive risk of thrombosis after the Janssen vaccine resulted in a 10-day suspension (April 13 to April 23) of its administration in April 2021. The excessive reporting risk of GBS after Janssen vaccine was also reported by several studies^11,12^. For shingles, the VAERS data suggested that the 1st shot of Moderna vaccine was associated with the highest reporting risk, followed by the 2nd shot of Moderna vaccine. For myocarditis, higher reporting risks was observed for the second dose of Pfizer and Moderna vaccines. There are some post-marketing studies on myocarditis after COVID-19 vaccination^13,14^ but no comparison between vaccines exists. For anaphylaxis higher reporting risks were observed for the first dose of Pfizer vaccine. One study^8^ showed that the incidence of anaphylaxis associated with the Pfizer vaccine is higher than other non-COVID vaccines, but no comparison among COVID-19 vaccines exist. There were no substantial differences among vaccines regarding risks of other AEs (Fig. 1). We emphasize that the rank consistency for local and systemic AEs adds credibility to the relative safety among multiple vaccines for the rare but severe AEs, and this is a unique contribution of our study.

In sensitivity analyses examining temporal changes on reporting risk we found decreasing risks of mild and common AEs such as pain and fever (Fig. S1a, b), and increasing risks of more severe and rare AEs such as thrombosis and GBS (Fig. S1c, d). The ranking of vaccines remained stable, and the correlations show consistency (i.e. correlation > 0) of the vaccines’ ranks between RCTs and VAERS, by using the VAERS reports as early as the first 2 months after administration. For more visualization see https://chongliang-luo.shinyapps.io/covid_vaers/, where the temporal changes on reporting risk are shown with user-specified age group, sex and AEs.

## Discussion

We analyzed COVID-19 vaccine safety data from published RCTs and the VAERS database. RCTs and VAERS rank COVID-19 vaccines similarly by risk of AE, supporting the validity of VAERS for assessing relative AE risk in this context. To the extent we can extrapolate from AEs reported in both data sources to AEs reported only in VAERS, we can use VAERS to estimate the relative safety of the vaccines against serious side effects too rare to be measured effectively in RCTs, and in real time.

Our investigation has several strengths. First, our study investigated the evidence consistency by comparing safety signals across multi-source data. The consistency in evidence across datasets is an important component of evidence quality, which cannot be obtained using a single dataset. Second, severe AEs are better monitored by post-marketing data than RCTs. Our analysis using VAERS provides valuable evidence of the relative safety of the COVID-19 vaccines regarding severe AEs, and circumvents the reporting bias of the spontaneous reporting system.

Our study has several limitations. First, our analysis focuses on the comparison across vaccines, and evidence from RCTs and VAERS, but does not provide information on absolute incidences of AEs among the general population. Also, the risk calculations in RCTs and VAERS do not take into account background risks in the reporting populations. Second, as a spontaneous reporting system, reporting into VAERS varies over time and is subject to shifts in the population undergoing vaccination and to publicity stimulation, hence the inherent limitations/biases when using VAERS data for vaccine safety evaluation. For example, before vaccines were available to the general public, they were administered according to scheduled phases depending on age, occupation, and living situation; AEs and reporting practices may vary accordingly. These novel vaccines drew extraordinary public attention, especially when first administered, which may also affect reporting practices. Third, our study only includes three pivotal RCTs^1–4^ for the three vaccines but more trials may be included in the future. Furthermore, it lacks comparison with other safety systems such as the Biologics Effectiveness and Safety (BEST^15^), the Safety Platform for Emergency Vaccines (SPEAC^16^), the Clinical Immunization Safety Assessment (CISA^17^) project and the Vaccine Safety Datalink (VSD^18^). The current investigation is less robust unless more trials and safety surveillance databases are included in the comparison.

The validity of this study is largely dependent on the assumption that the reporting behavior to VAERS (i.e. reporting sensitivity^19^) is similar across different type of vaccines. This assumption is likely to be the case for Pfizer and Moderna vaccines, as they were authorized and became available at similar times. However, the Janssen vaccine was authorized about 3 months later and could have drawn less public attention. It is thus possible that people with only mild AEs after the Janssen vaccine may have chosen not to report to VAERS and as a result, the risks of more severe, systemic AEs is higher than those after Pfizer and Moderna vaccines. This could explain the inconsistency of the rank of Janssen vaccine between RCT and VAERS regarding systemic AEs in Table 2. We have previously seen such an example in our evaluation of GBS as a potential post-vaccination effect after influenza vaccines^6^, where people who reported GBS tend not to report mild AEs, such as pain, erythema and swelling etc.

Post-market surveillance is essential for continuously evaluating and monitoring vaccine safety. In addition to VAERS, other active surveillance databases such as BEST^15^, SPEAC^16^, CISA^17^ and VSD^18^ have been used for evaluating the safety of COVID-19 vaccines^7,20–22^. In general, data collected by surveys^23,24^ or social media platforms^25,26^ could also provide evidences for vaccine safety or hesitancy. Despite the limitation of being a passive reporting system, VAERS, with proper statistical analyses, still provides valuable information about vaccine safety, especially in the early phase of general population administration and vaccine surveillance. For example, in the first several months (Dec 2020–April 2021) of the Pfizer and Moderna COVID-19 vaccine administration, VAERS-based studies^5,8–10^ emerged to generate safety signals for anaphylaxis, sudden hearing loss and cerebral venous sinus thrombosis. These studies contribute unique time-sensitive real-world evidence on safety signals that were examined rigorously by later randomized studies. Our study provides a novel method utilizing the publicly available RCT and VAERS data for monitoring the safety of COVID-19 vaccines. It is reassuring that our analyses based on the VAERS data produced evidence consistent with existing RCTs, and demonstrated the ability to identify rare AEs that were not recognized in the RCTs. Our analysis can be applied to the post-market surveillance of ongoing (e.g. COVID-19 vaccine boost dose and adolescent dose) and future vaccine administration using VAERS, especially for the severe AEs. The website https://chongliang-luo.shinyapps.io/covid_vaers/ will be continuously updated for timely monitoring the COVID-19 vaccines in the future.

## Methods

### Data sources

We collected vaccine safety data from published RCTs for the three vaccines authorized in the U.S. For all RCTs^1–4^, local AEs (i.e. pain, erythema and swelling) and systemic AEs (i.e. headache, fever, chill, fatigue, nausea/vomiting, arthralgia/joint pain, and myalgia/muscle pain) were assessed within seven days after vaccination.

We also used data from VAERS^27–29^ to seek post-marketing evidence on the safety of the three vaccines. The VAERS data is publicly available and no institutional approval is required. The analyses were performed in accordance with VAERS guidelines and regulations^27^. We extracted AE reports after COVID-19 vaccination up to September 17, 2021. Detailed definitions of the AEs in MedDRA preferred term (PT) level are listed in Table S1. We grouped some of the similar PT terms to their Standardized MedDRA Queries (SMQs)^30^. Reports with co-administration with other vaccines, or unknown manufacturer of COVID-19 vaccine, or invalid symptoms (e.g. “no adverse events”) were excluded. To be consistent with the RCTs, we used VAERS reports with age 18 + (age 16 + for the Pfizer vaccine), and onset time within 7 days after vaccination for the solicited AEs (i.e. local and systemic AEs). For the unsolicited AEs (i.e. other rare but severe AEs including shingles, hearing impairment including tinnitus, thrombosis, facial paralysis including Bell’s palsy, anaphylaxis, pulmonary embolism, myocarditis, and GBS), we used VAERS reports with onset time within 42 days after vaccination. Analysis was also repeated for age groups, i.e. young and elderly populations. We used the age cutoffs from the original trials (i.e. 55 for Pfizer trial, 65 for Moderna trial and 60 for Janssen trial) for the RCTs, and 60 for VAERS.

### Statistical analysis

**Figure 2:**
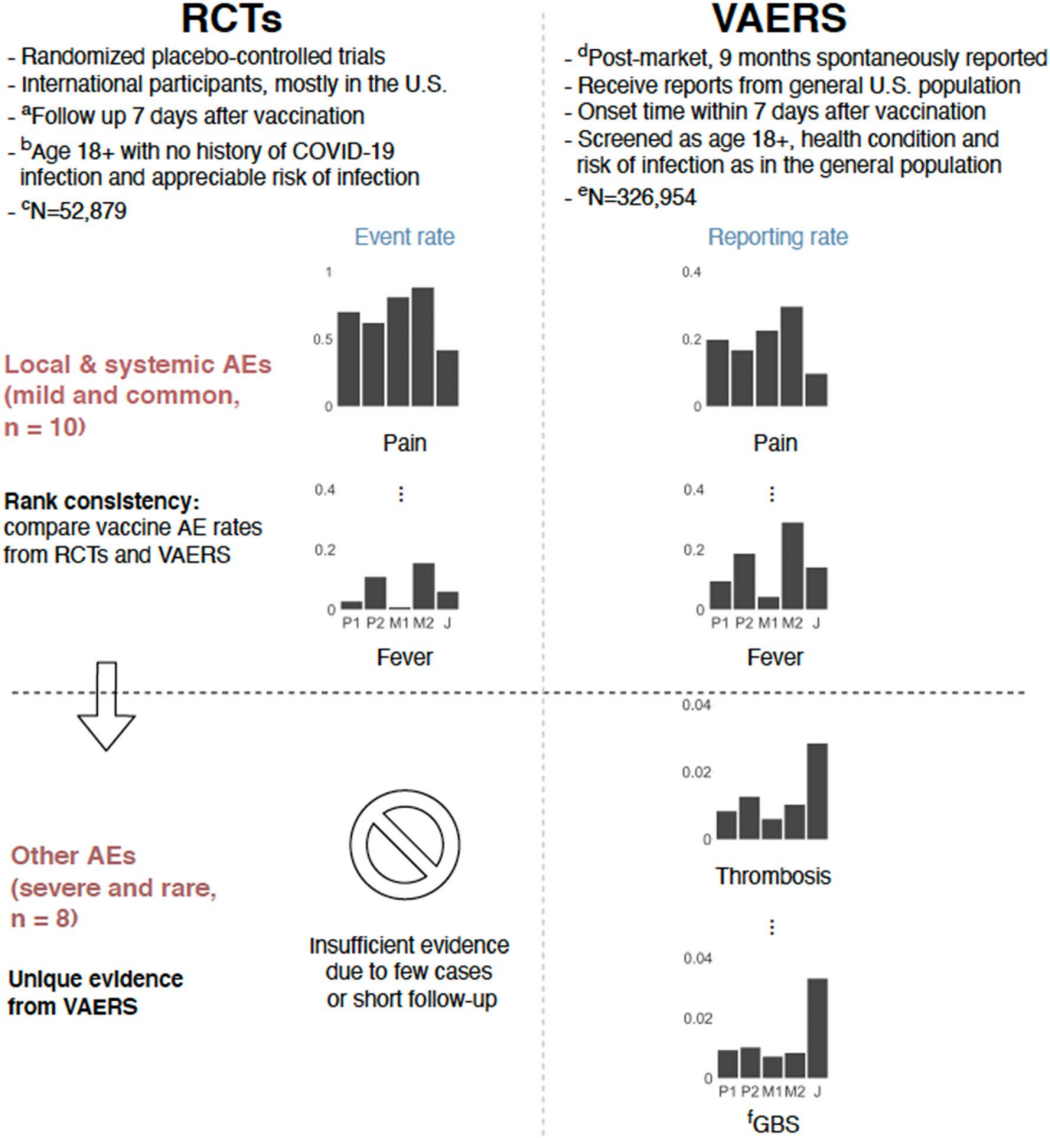
COVID-19 vaccine safety study from three randomized controlled trials^1–4^ (RCTs) and the CDC vaccine adverse event reporting system (VAERS). Five groups are compared, i.e. Pfizer-1st (P1), Pfizer-2nd (P2), Moderna-1st (M1), Moderna-2nd (M2) and Janssen (J). Rank consistency is shown between RCTs and VAERS regarding local and systemic adverse events (AEs), including pain, erythema, swelling, headache, fever, chill, fatigue, nausea/vomiting, arthralgia and myalgia. The evidence is extended to study the other severe and rare AEs including shingles, hearing impairment, thrombosis, facial paralysis, anaphylaxis, pulmonary embolism, myocarditis, and GBS using VAERS. ^a^Follow up 7 days for solicited local and systemic AEs, and 1 month for unsolicited AEs; ^b^age 16 + for the Pfizer trial, and no exclusion of participants with history of COVID-19 infection for the Janssen trial; ^c^sample size N = 9839, 9839, 15,168, 14,677 and 3356 for P1, P2, M1, M2 and J, respectively; ^d^reported to VAERS from Dec. 2020 to Sep. 2021; ^e^N = 83,877, 57,431, 101,383, 54,386 and 29,877 for P1, P2, M1, M2 and J respectively; ^f^*GBS* Guillain–Barré syndrome.

The vaccines and doses were grouped in 5 categories, i.e. Pfizer-1st (P1), Pfizer-2nd (P2), Moderna-1st (M1), Moderna-2nd (M2) and Janssen (J). Figure 2 illustrates the overall rationale of our statistical analysis plan using data from RCTs and VAERS. We calculated AE risks for each vaccine type and dose in VAERS. Since the AEs are self-reported in the VAERS database, AE risks are not directly comparable to event risks in the RCTs. However, a unique opportunity in our investigation of VAERS data on COVID-19 vaccines is that the Pfizer and Moderna vaccines were released around the same time and both have received similar public attention. Thus, it is reasonable to assume that the reporting behavior of AEs is not strongly dependent on the type of vaccine received by the vaccinee. Under this assumption, despite the VAERS data being subject to reporting bias, a similar reporting risk applies to both vaccinees, hence the ranks of AE reporting risks across vaccines calculated from VAERS are meaningful statistics, which can be cross-compared with the ranks of AE risks across vaccines calculated from RCTs.

Given the above rationale, we compared AE ranking by vaccine in both the VAERS and RCT databases, and evaluated their consistency. The rank consistency was measured by the Spearman’s rank correlation coefficient $\rho$^31^. Spearman’s $\rho$ ranges from − 1 to 1 and a positive value indicates consistency of the two ranks. This rank correlation measure is commonly used to assess agreement between two rating approaches.

Finally, based on the consistency between RCTs and VAERS, we further ranked the vaccines regarding the risks of other rare but severe AEs (i.e. shingles, hearing impairment including tinnitus, thrombosis, facial paralysis including Bell’s palsy, anaphylaxis, pulmonary embolism, myocarditis, and GBS) in VAERS.

## Supplementary Information

Supplementary Information 1.

Supplementary Information 2.

## Data Availability

The RCT data analyzed in this study are available from the published papers^1–4^; the VAERS data are publically available from https://vaers.hhs.gov/data.html.
