# Supplementary material for: Comparability of clinical trials and spontaneous reporting data regarding COVID-19 vaccine safety
Source: Sci Rep. 2022 Jun 29;12:10946. doi: 10.1038/s41598-022-13809-7 (PMC9243073; doi:10.1038/s41598-022-13809-7)
Supplement: Supplementary file 2 — Supplementary Information 2. [file 41598_2022_13809_MOESM2_ESM.docx]

## Supplementary Materials for “Comparability of Clinical Trial and Spontaneous Reporting Data Regarding COVID-19 Vaccine Safety”

Table S1. Risks (%) of various AEs for young recipients related to the three COVID-19 vaccines (and doses) in the U.S. by randomized clinical trials (RCT) or vaccine adverse event reporting system (VAERS) data. Five groups of vaccines are compared, i.e. Pfizer-1^st^ (P1), Pfizer-2^nd^ (P2), Moderna-1^st^ (M1), Moderna-2^nd^ (M2) and Janssen (J). Age 16-54 for Pfizer trial, 18-64 for Moderna trial, 18-59 for Janssen trial, and 18-59 for VAERS. A rank correlation greater than 0 means the rank of vaccines by RCT and VAERS are consistent. The “other AEs” are not explicitly evaluated in RCTs.

| Adverse events (AE) | Data source | AE risks (%) by vaccine groups | | | | | Rank | | | | | | Rank correlation  (Spearman’s $\rho$) |
| --- | --- | --- | --- | --- | --- | --- | --- | --- | --- | --- | --- | --- | --- |
|  |  | P1 | P2 | M1 | M2 | J |  |  | risk increase | | |  |  |
| **Local AEs (joint evidence generated from RCTs and VAERS)** | | | | | | | | | | | | | |
| Pain | RCT | 83 | 78 | 86.9 | 89.9 | 57.0 | J | P2 | P1 | M1 | M2 | | 0.80 |
|  | VAERS | 7.4 | 8.4 | 12.9 | 11.2 | 7.3 | J | P1 | P2 | M2 | M1 | |  |
| Erythema | RCT | 5 | 6 | 3 | 8.9 | 8.0 | M1 | P1 | P2 | J | M2 | | -0.40 |
|  | VAERS | 11.2 | 10.1 | 24.7 | 17.8 | 7.5 | J | P2 | P1 | M2 | M1 | |  |
| Swelling | RCT | 6 | 6 | 6.7 | 12.6 | 7.0 | J | M1 | P1 | P2 | M2 | | 0.15 |
|  | VAERS | 9.5 | 9 | 17.8 | 13.5 | 6.6 | J | P2 | P1 | M2 | M1 | |  |
| **Systemic AEs (joint evidence generated from RCTs and VAERS)** | | | | | | | | | | | | | |
| Headache | RCT | 42 | 52 | 35.3 | 62.8 | 44.0 | M1 | P1 | J | P2 | M2 | | 0.60 |
|  | VAERS | 17.8 | 27.5 | 19.8 | 30.1 | 32.3 | P1 | M1 | P2 | M2 | J | |  |
| Fever | RCT | 4 | 16 | 0.9 | 17.4 | 12.0 | M1 | P1 | J | P2 | M2 | | 0.80 |
|  | VAERS | 9.8 | 25.9 | 14.9 | 32 | 28.0 | P1 | M1 | P2 | J | M2 | |  |
| Chills | RCT | 14 | 35 | 9.2 | 48.6 |  | M1 | P1 | P2 | M2 | J | | 0.80 |
|  | VAERS | 9.5 | 23.1 | 13.7 | 28.4 | 26.0 | P1 | M1 | P2 | J | M2 | |  |
| Fatigue | RCT | 47 | 59 | 38.4 | 67.6 | 44.0 | M1 | J | P1 | P2 | M2 | | 0.70 |
|  | VAERS | 14.9 | 21.9 | 16.2 | 23.5 | 21.4 | P1 | M1 | J | P2 | M2 | |  |
| Nausea  /Vomiting | RCT | 1 | 2 | 9.4 | 21.4 | 15.0 | P1 | P2 | M1 | J | M2 | | 0.80 |
|  | VAERS | 14.2 | 17 | 14.7 | 19.4 | 21.8 | P1 | M1 | P2 | M2 | J | |  |
| Arthralgia  (joint pain) | RCT | 11 | 22 | 16.6 | 45.5 |  | P1 | M1 | P2 | M2 | J | | 1 |
|  | VAERS | 8.2 | 11.7 | 8.7 | 11.8 | 9.9 | P1 | M1 | J | P2 | M2 | |  |
| Myalgia  (muscle pain) | RCT | 21 | 37 | 23.7 | 61.6 | 39.0 | P1 | M1 | J | P2 | M2 | | 0.82 |
|  | VAERS | 7.2 | 11.5 | 8.7 | 11.5 | 12.4 | P1 | M1 | P2 | M2 | J | |  |
| **Other AEs (unique evidence generated from VAERS)** | | | | | | | | | | | | | |
| Shingles | VAERS | 7.8 | 6.6 | 15.2 | 9.9 | 5.1 | J | P2 | P1 | M2 | M1 | |  |
| Hearing impairment | VAERS | 3.1 | 2.7 | 1.9 | 2.5 | 2.8 | M1 | M2 | P2 | J | P1 | | N/A |
| Thrombosis | VAERS | 0.7 | 0.9 | 0.5 | 0.7 | 2.1 | M1 | P1 | M2 | P2 | J | |  |
| Facial paralysis | VAERS | 1 | 0.7 | 0.6 | 0.6 | 0.8 | M2 | M1 | P2 | J | P1 | |  |
| Anaphylaxis | VAERS | 0.6 | 0.3 | 0.4 | 0.3 | 0.2 | J | M2 | P2 | M1 | P1 | |  |
| Pulmonary embolism | VAERS | 0.2 | 0.3 | 0.2 | 0.3 | 0.7 | M1 | P1 | P2 | M2 | J | |  |
| Myocarditis | VAERS | 0.3 | 0.9 | 0.2 | 0.8 | 0.2 | M1 | J | P1 | M2 | P2 | |  |
| GBS | VAERS | 0.1 | 0.1 | 0.1 | 0.1 | 0.3 | M1 | M2 | P1 | P2 | J | |  |

Table S2. Risks (%) of various AEs for elderly recipients related to the three COVID-19 vaccines (and doses) in the U.S. by randomized clinical trials (RCT) or vaccine adverse event reporting system (VAERS) data. Five groups of vaccines are compared, i.e. Pfizer-1^st^ (P1), Pfizer-2^nd^ (P2), Moderna-1^st^ (M1), Moderna-2^nd^ (M2) and Janssen (J). Age 55+ for Pfizer trial, 65+ for Moderna trial, 60+ for Janssen trial, and 60+ for VAERS. A rank correlation greater than 0 means the rank of vaccines by RCT and VAERS are consistent. The “other AEs” are not explicitly evaluated in RCTs.

| Adverse events (AE) | Data source | AE risks (%) by vaccine groups | | | | | Rank | | | | | | Rank correlation  (Spearman’s $\rho$) |
| --- | --- | --- | --- | --- | --- | --- | --- | --- | --- | --- | --- | --- | --- |
|  |  | P1 | P2 | M1 | M2 | J |  |  | risk increase | | |  |  |
| **Local AEs (joint evidence generated from RCTs and VAERS)** | | | | | | | | | | | | | |
| Pain | RCT | 71 | 66 | 74 | 83.2 | 32.0 | J | P2 | P1 | M1 | M2 | | 0.90 |
|  | VAERS | 7.1 | 6.7 | 12.6 | 10 | 5.6 | J | P1 | P2 | M2 | M1 | |  |
| Erythema | RCT | 5 | 7 | 2.3 | 7.5 | 4.0 | M1 | P1 | P2 | J | M2 | | 0 |
|  | VAERS | 11.9 | 12 | 26.4 | 19.3 | 9.0 | J | P2 | P1 | M2 | M1 | |  |
| Swelling | RCT | 7 | 7 | 4.4 | 10.8 | 3.0 | J | M1 | P1 | P2 | M2 | | 0.36 |
|  | VAERS | 9.6 | 8.9 | 16.6 | 12.4 | 7.6 | J | P2 | P1 | M2 | M1 | |  |
| **Systemic AEs (joint evidence generated from RCTs and VAERS)** | | | | | | | | | | | | | |
| Headache | RCT | 25 | 39 | 24.5 | 46.2 | 30.0 | M1 | P1 | J | P2 | M2 | | 0.60 |
|  | VAERS | 17.3 | 21.9 | 18 | 24.3 | 25.6 | P1 | M1 | P2 | M2 | J | |  |
| Fever | RCT | 1 | 11 | 0.3 | 10 | 3.0 | M1 | P1 | J | P2 | M2 | | 0.80 |
|  | VAERS | 9.5 | 17.7 | 13.8 | 24.9 | 16.9 | P1 | M1 | P2 | J | M2 | |  |
| Chills | RCT | 6 | 23 | 5.4 | 30.9 |  | M1 | P1 | P2 | M2 | J | | 0.80 |
|  | VAERS | 10.6 | 17.7 | 13.9 | 23.4 | 17.4 | P1 | M1 | P2 | J | M2 | |  |
| Fatigue | RCT | 34 | 51 | 33.3 | 58.3 | 29.0 | M1 | J | P1 | P2 | M2 | | 0.30 |
|  | VAERS | 14.8 | 18.6 | 16 | 22.5 | 19.0 | P1 | M1 | J | P2 | M2 | |  |
| Nausea  /Vomiting | RCT | 0 | 1 | 5.2 | 11.8 | 12.0 | P1 | P2 | M1 | J | M2 | | 0.82 |
|  | VAERS | 12.4 | 13.8 | 12.4 | 15.4 | 16.3 | P1 | M1 | P2 | M2 | J | |  |
| Arthralgia  (joint pain) | RCT | 9 | 19 | 16.4 | 35 |  | P1 | M1 | P2 | M2 | J | | 0.74 |
|  | VAERS | 10.4 | 12.1 | 9.6 | 12.1 | 10.9 | P1 | M1 | J | P2 | M2 | |  |
| Myalgia  (muscle pain) | RCT | 14 | 29 | 19.7 | 47.1 | 24.0 | P1 | M1 | J | P2 | M2 | | 0.90 |
|  | VAERS | 6.9 | 9.1 | 8.8 | 10.4 | 9.7 | P1 | M1 | P2 | M2 | J | |  |
| **Other AEs (unique evidence generated from VAERS)** | | | | | | | | | | | | | |
| Shingles | VAERS | 7.8 | 7.5 | 15.9 | 10.4 | 6.2 | J | P2 | P1 | M2 | M1 | |  |
| Hearing impairment | VAERS | 3.2 | 3.3 | 1.6 | 2.6 | 3 | M1 | M2 | J | P1 | P2 | | N/A |
| Thrombosis | VAERS | 1.1 | 1.7 | 0.6 | 1.2 | 5.2 | M1 | P1 | M2 | P2 | J | |  |
| Facial paralysis | VAERS | 1.2 | 1 | 0.7 | 0.6 | 1.2 | M2 | M1 | P2 | J | P1 | |  |
| Anaphylaxis | VAERS | 0.4 | 0.2 | 0.2 | 0.1 | 0.2 | M2 | J | P2 | M1 | P1 | |  |
| Pulmonary embolism | VAERS | 0.5 | 0.9 | 0.4 | 0.7 | 2.1 | M1 | P1 | M2 | P2 | J | |  |
| Myocarditis | VAERS | 0.2 | 0.4 | 0.1 | 0.2 | 0.3 | M1 | P1 | M2 | J | P2 | |  |
| GBS | VAERS | 0.1 | 0.2 | 0.1 | 0.1 | 0.7 | M1 | M2 | P1 | P2 | J | |  |

## Temporal analysis

## The reported risks in VAERS also show temporal trend depending on the severity of the AEs, which is visualized in the Supplementary Figure S1. There is a clear decreasing trend for the risks of mild and common AEs such as pain and fever (Figure S1a, b), and an increasing trend for the risks of more severe and rare AEs such as thrombosis and GBS (Figure S1c, d). This is likely due to the spontaneous reporting bias, i.e. only severe AEs are mentioned in VAERS reports when the COVID-19 vaccines become more familiar. In fact, relatively fewer people reported to VAERS at later time. This might because the side effects of COVID-19 vaccines become more familiar to people, especially for the mild and common AEs. One thing worth noticing from Figure S1 is, the rank of vaccines remains temporarily stable, though the AE risks changes over time. The correlations show consistency (i.e. correlation > 0) of the vaccines’ ranks between RCTs and VAERS, by using the VAERS reports as early as the first 2 months after administration. This demonstrates the potential for monitoring rare AEs and comparing multiple vaccines in a timely manner. For reproducibility of our analyses, we have implemented the comparison of vaccines within each given age and sex subgroup using the most updated VAERS data at https://chongliang-luo.shinyapps.io/covid_vaers/, which allows customized AE symptom in addition to the set of AEs studied in this paper. This could be used for future surveillance, e.g. the boosting shot and the vaccination of children.

##
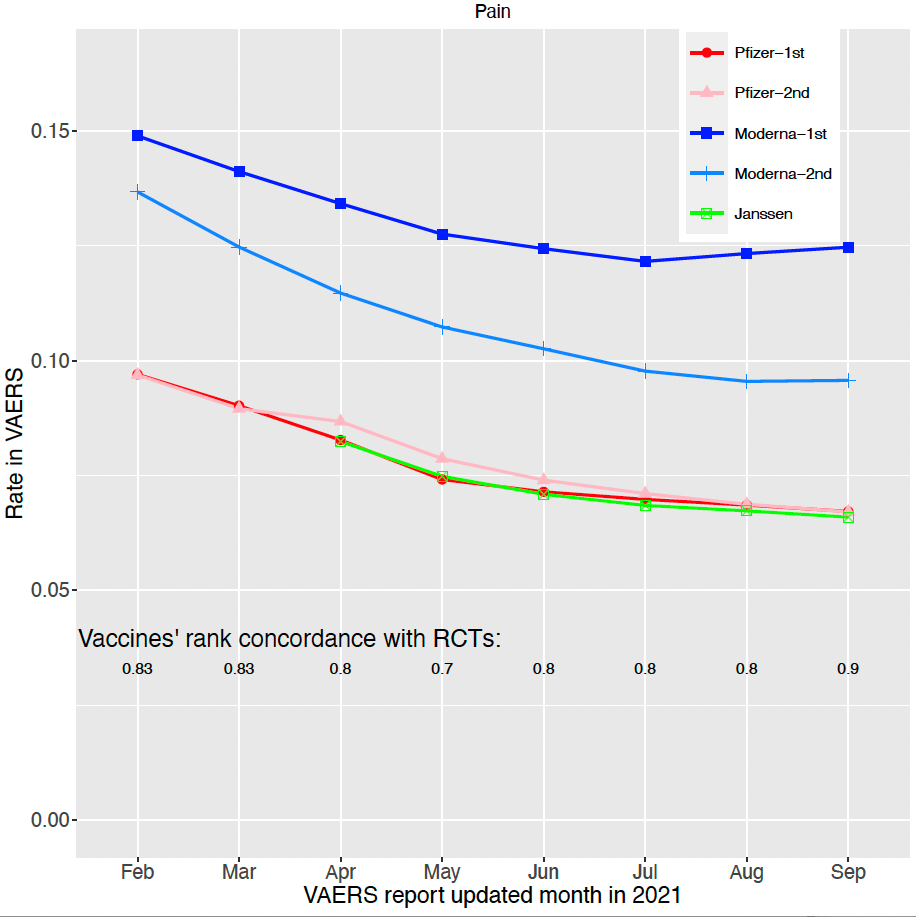

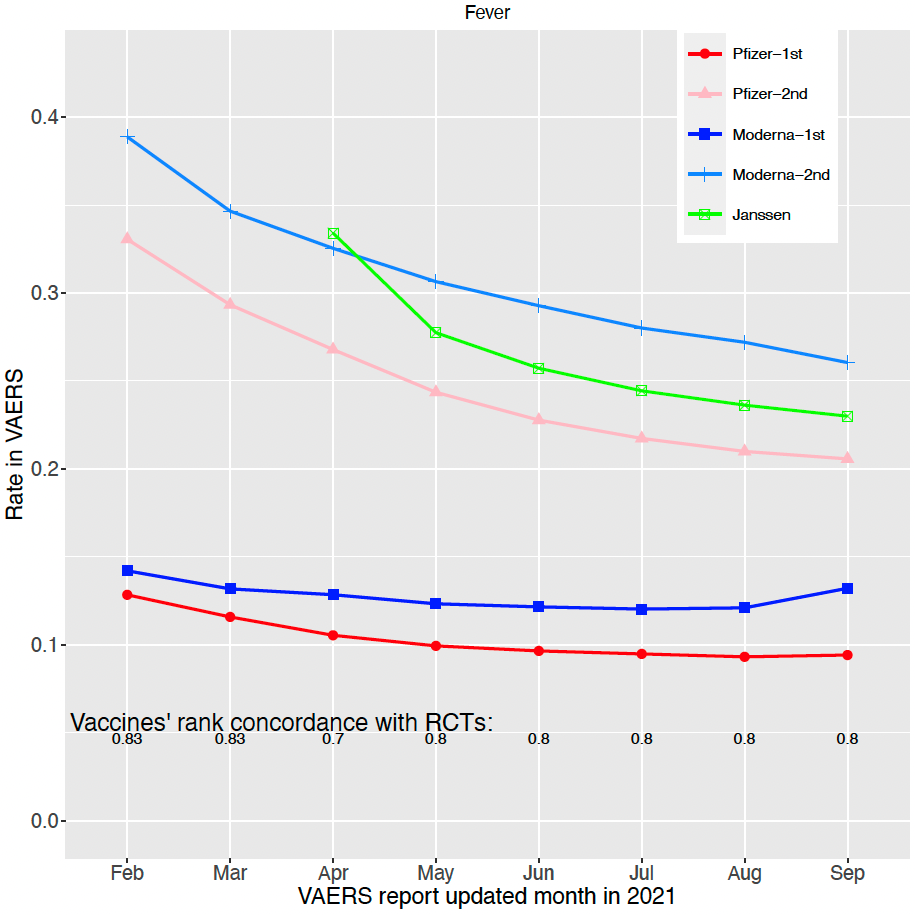


## (a) (b)

##
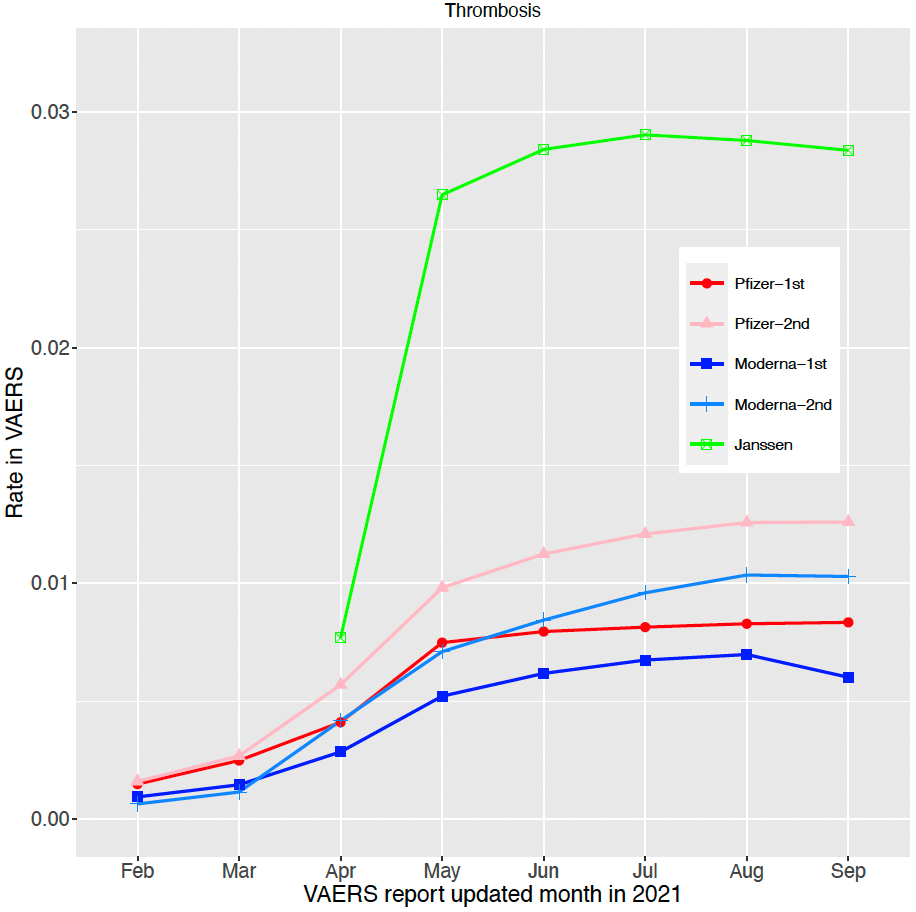

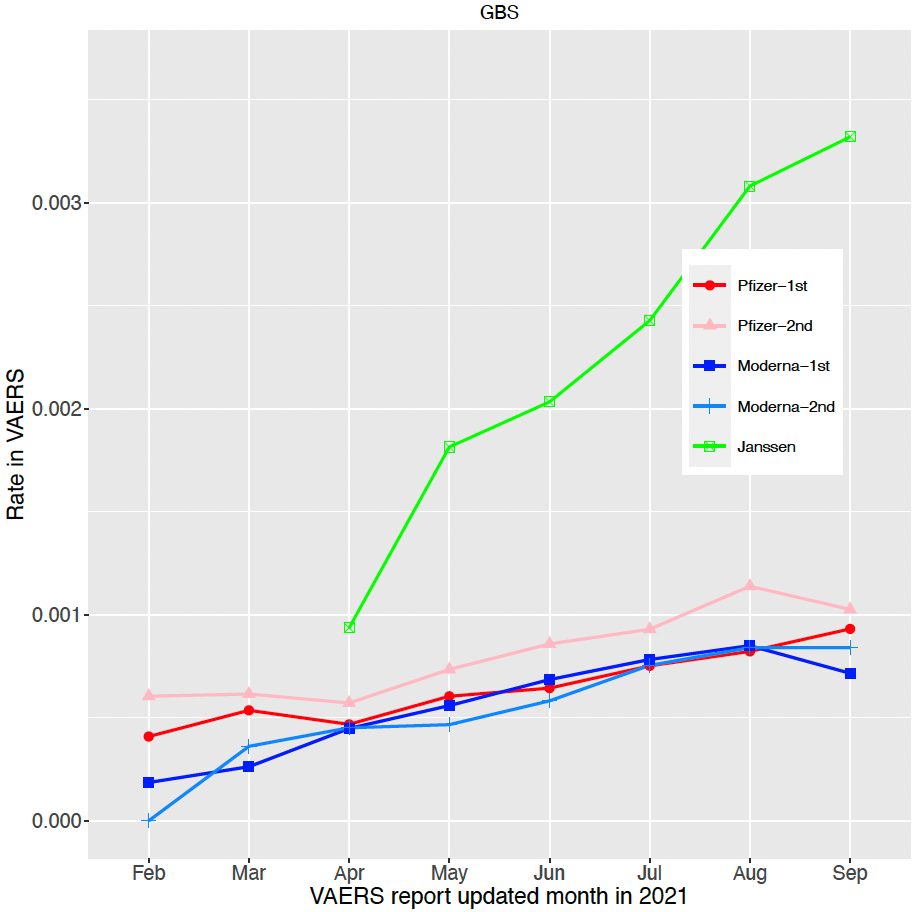


(c) (d)

Figure S1. Cumulative risks of specific AEs after COVID-19 vaccines in VAERS (reports updated in the middle of each month). Pain and fever are typical mild and common AEs, while thrombosis and GBS are typical severe and rare AEs.

Table S3. Adverse events defined in MedDRA PT terms from the VAERS database.

| Adverse events | Category | MedDRA PT |
| --- | --- | --- |
| Pain | Local Events | Injection site pain\|Vaccination site pain\|Administration site pain |
| Erythema | Local Events | Erythema\|Rash\|Redness |
| Swelling | Local Events | Swelling |
| Headache | SystemicEvents | Headache |
| Fever | SystemicEvents | Pyrexia\|Hyperpyrexia\|Sweating fever |
| Chills | SystemicEvents | Chills |
| Fatigue | SystemicEvents | Fatigue |
| Nausea / Vomiting | SystemicEvents | Nausea\|Vomiting\|Vomiting projectile\|Discoloured vomit\|Cyclic vomiting syndrome |
| Arthralgia (joint pain) | SystemicEvents | SMQ^29^: Arthritis |
| Myalgia (muscle pain) | SystemicEvents | Myalgia\|Muscle |
| Shingles | Other AE | Shingles\|Rash |
| Hearing impairment | Other AE | Hearing loss\|Tinnitus\|Deaf\|Hypoacusis\|Hyperacusis |
| Thrombosis | Other AE | Thrombosis |
| Facial paralysis | Other AE | Facial paralysis\|Bell's palsy |
| Anaphylaxis | Other AE | Anaphylact |
| Pulmonary embolism | Other AE | Pulmonary embolism |
| Myocarditis | Other AE | Myocarditis\|Heart inflammation\|Pericarditis\|Cardiomyopathy |
| GBS | Other AE | Guillain-Barre syndrome\|Miller-Fisher syndrome\|Demyelinating polyneuropathy |
